# Monkeying around with venom: an increased resistance to α-neurotoxins supports an evolutionary arms race between Afro-Asian primates and sympatric cobras

**DOI:** 10.1186/s12915-021-01195-x

**Published:** 2021-11-25

**Authors:** Richard J. Harris, K. Anne-Isola Nekaris, Bryan G. Fry

**Affiliations:** 1 Venom Evolution Lab, University of Queensland, Biological Sciences, St. Lucia, Brisbane, 4072 Australia; 2 Nocturnal Primate Research Group, Department of Social Sciences, Oxford Brookes University, Oxford, OX3 0BP UK

**Keywords:** Venom, Evolution, Resistance, Primate, Neurotoxin

## Abstract

**Background:**

Snakes and primates have a multi-layered coevolutionary history as predators, prey, and competitors with each other. Previous work has explored the Snake Detection Theory (SDT), which focuses on the role of snakes as predators of primates and argues that snakes have exerted a selection pressure for the origin of primates’ visual systems, a trait that sets primates apart from other mammals. However, primates also attack and kill snakes and so snakes must simultaneously avoid primates. This factor has been recently highlighted in regard to the movement of hominins into new geographic ranges potentially exerting a selection pressure leading to the evolution of spitting in cobras on three independent occasions.

**Results:**

Here, we provide further evidence of coevolution between primates and snakes, whereby through frequent encounters and reciprocal antagonism with large, diurnally active neurotoxic elapid snakes, Afro-Asian primates have evolved an increased resistance to α-neurotoxins, which are toxins that target the nicotinic acetylcholine receptors. In contrast, such resistance is not found in Lemuriformes in Madagascar, where venomous snakes are absent, or in Platyrrhini in the Americas, where encounters with neurotoxic elapids are unlikely since they are relatively small, fossorial, and nocturnal. Within the Afro-Asian primates, the increased resistance toward the neurotoxins was significantly amplified in the last common ancestor of chimpanzees, gorillas, and humans (clade Homininae). Comparative testing of venoms from Afro-Asian and American elapid snakes revealed an increase in α-neurotoxin resistance across Afro-Asian primates, which was likely selected against cobra venoms. Through structure-activity studies using native and mutant mimotopes of the α-1 nAChR receptor orthosteric site (loop C), we identified the specific amino acids responsible for conferring this increased level of resistance in hominine primates to the α-neurotoxins in cobra venom.

**Conclusion:**

We have discovered a pattern of primate susceptibility toward α-neurotoxins that supports the theory of a reciprocal coevolutionary arms-race between venomous snakes and primates.

**Supplementary Information:**

The online version contains supplementary material available at 10.1186/s12915-021-01195-x.

## Background

Snakes and primates have a sophisticated coevolutionary history as predators, prey, and competitors with each other. Prior work has focused on two distinct areas: (1) the Snake Detection Theory (SDT), in which encounters with snakes have shaped primate evolution, and (2) how primate diversification has shaped the evolution of venomous snakes’ defensive behaviors and morphological innovations.

The SDT of primate evolution encapsulates coevolutionary interactions between primates and snakes that have shaped primate neurobiology, psychology, and physiology [1, 2]. This theory proposes that constricting snakes selected for changes in visual systems that led to the differentiation of primates as a separate order of mammals. It also suggests that further visual system expansion later evolved in anthropoids, including Catarrhini (African/Asian monkeys, apes, and humans) and Platyrrhini (American monkeys), as a response to the evolution of venomous snakes in the families Elapidae and Viperidae [1, 2]. Predation by venomous snakes would become rarer events as primate body size increased, but defensive envenomation by these snakes would still exert a strong selection pressure, according to this theory. In support of the SDT, a visual bias leading to faster or more reliable detection of snakes than to other animals/objects has been found in those primates tested, i.e., catarrhines, including *Homo sapiens* [3–7]. However, the SDT suggests that rapid and reliable detection might be variable among primates because visual adaptations differ among major lineages and coincide with their evolutionary co-existence with venomous snakes. For example, primates of the suborder Strepsirrhini (Lorisiformes, Lemuriformes) have poorer central vision and acuity systems than the suborder Haplorhini (Tarsiiformes, Platyrrhini, and Catarrhini) because they do not have a true fovea [1, 2]. Lemuriformes colonized Madagascar between 50 and 65 MA [8], likely before the evolution of vipers ~ 50 MA [9], but certainly before their pan Afro-Asian radiation, and long before the evolution of elapid snakes and their characteristic neurotoxic venom ~ 37 MA [10]. Madagascar has remained devoid of venomous snakes; thus, the SDT proposes that there has never been a selection pressure on this group to evolve survival adaptations against venomous snakes. Although platyrrhines and catarrhines are anthropoid primates, which are thought to have evolved in Asia or Africa, platyrrhines have more variable visual systems than catarrhines [1, 2]. The platyrrhines diverged from other anthropoids after they dispersed to South America ~ 35 MA [11], long before the arrival of venomous snakes in South America between 3 and 25 MA, based on both molecular data and the formation of the Panamanian land bridge [9, 12–14]. Thus, unlike catarrhines, platyrrhines have had an interrupted evolutionary time scale with venomous snakes [1, 2]. Most platyrrhines are highly arboreal, while the recently sympatric venomous snakes are mostly terrestrial, with neurotoxic elapid species in particular being fossorial and crepuscular/nocturnal. Thus, selection pressures from venomous snakes are also expected to be weaker on platyrrhines than on catarrhines.

Conversely, there is evidence that venomous elapids have evolved some adaptations in response to primate diversification. Spitting in cobras has evolved on three convergent occasions (once in the genus *Hemachatus* and on two independent occasions within *Naja*) as a defensive trait that causes intense ocular pain and inflammation, which might have evolved in response to unique behavioral adaptations by anthropoids, particularly early hominines [15]. Predation events by natural predators (e.g., birds, mammals, and other reptiles) of spitting cobras often occur. Thus, it seems that spitting is not an effective method against most of their natural predators [15]. Intriguingly, the emergence of spitting in *Hemachatus* (< 17 MA), the African spitting *Naja* (~ 6.7 MA), and the Asian spitting *Naja* (~ 2.5 MA) [10], all coincided with the diverse emergence and radiations of terrestrial dwelling apes, including early hominines [16, 17], all of which occurred in habitats also frequented by largely terrestrial spitting cobras [18]. These hominines showed a myriad of features indicating a tendency toward upright bipedal movement and increased precision and opposability of the hands and thumbs. It has been inferred that the last common ancestors of chimpanzees and humans already used wood and stone tools and had the ability to hunt vertebrates [17]. Furthermore, terrestrial dwelling apes, through their locomotor style, foraging habits, and group movements, were more likely than other terrestrial taxa to come into frequent contact with venomous snakes [19]. The emergence of Asian spitting cobras at ~ 2.5 MA [10] coincided with the appearance of *Homo erectus* in Asia (~ 1.8 MA), the latter of which is characterized by advancements in stone tool technology [20]. Primates in general show overt reactions to snakes, including snake-specific alarm calls, and may mob snakes with improvised tools and projectiles [21–23]. Thus, the use of projectile weaponry in primates, particularly in anthropoids that stand upright when using such weapons, would provide a selection pressure on snakes to evolve a ranged defense such as spitting venom at the eyes of upright primates. Additionally, the evolution of injury-inducing defensive cytotoxins is also correlated with that of defensive hooding displays and aposematic marking in African and Asian cobras [24]. The evolution of defensive cytotoxins also preceding spitting suggests that a high selection pressure might have been exerted upon cobras for defense against primates before the emergence of spitting when hominins evolved bipedalism. Hence, there is some fundamental evidence to consider that reciprocal coevolution occurred between primates and snakes, particularly venomous snakes, driving novel traits in both taxa.

Non-primate mammals that co-exist with venomous snakes have some resistance toward snake venom [25–30], while populations absent of venomous snakes do not [31]. A prominently evolved resistance mechanism is seen at the orthosteric site (acetylcholine (ACh) binding region loop C [32–34]) of the α-1 nicotinic acetylcholine receptor (nAChR) by which specific amino acid residues reduce the binding of α-neurotoxins (such as three-finger toxins; 3FTxs) found within some snakes venoms, particularly that of Elapidae [35, 36]. The α-1 nAChR is located at the neuromuscular junction, ultimately controlling muscle contraction and function, and thus is a prime target for many toxins that cause paralysis, such as predatory venoms. The α-1 nAChR orthosteric site of humans has been shown to be much less susceptible to binding of α-neurotoxins [37–40] in contrast to the same site binding of other taxa [41–45]. Thus, while neurotoxic effects of elapid venoms may be a lethal outcome in some human envenomations (but not always), it is possible that in these instances, binding allosterically (to a receptor region other than the orthosteric site) might be a more derived method [46], rather than binding to the orthosteric site.

Despite this increased resistance in *H. sapiens* and given the mounting evidence in support of their coevolution with venomous snakes, no studies have attempted to investigate how α-neurotoxin binding might affect a wide range of primate species. Afro-Asian primates co-exist with large, abundant, diurnally active cobras that readily defend themselves, leading to death. Thus, we set out to test the hypothesis that if resistance to these paralytic toxins evolved, it would be present in Afro-Asian primates and lacking in primates from other regions that either lack venomous snakes (Madagascar) or where neurotoxic elapid snakes are small, fossorial, and crepuscular/nocturnal and encounters are rare (the Americas).

## Results

Investigations into the publicly available α-1 nAChR sequences of a range of primate species revealed that the orthosteric site loop C region of *H. sapiens* is conserved across the Homininae (chimpanzees, gorillas, humans), while other primates such as Cercopithecidae (African/Asian monkeys), Galagidae (bushbabies), Lemuriformes (lemurs), Platyrrhini (American monkeys) and Ponginae (orangutans) all have clade-specific orthosteric sequences (Table S1). Given these differences among primate clades and the theory that primates and snakes might have coevolved certain traits through reciprocal antagonism, we tested if there were any differences in α-neurotoxin binding between primate lineages, particularly given evidence that *H. sapiens* nAChRs are less susceptible to α-neurotoxins [37–40]. To assess these differences, we tested seven venoms that are rich in α-neurotoxins (3FTxs): five from species of cobra (*Naja* spp.) that inhabit Africa (*N. haje*, *N. mossambica*, and *N. nubiae*) and Asia (*N. kaouthia* and *N. siamensis*) and two species of *Micrurus* from South America (*M. browni* and *M. corallinus*). We further categorized the cobras into spitting (*N. mossmabica*, *N. nubiae*, and *N. siamensis*) and non-spitting (*N. haje* and *N. kaouthia*) species. We tested the binding of these venoms upon mimotopes that represent the orthosteric site loop C region of α-1 nAChRs from different primate clades (Table S1). We utilized a biolayer interferometry (BLI) assay, which has been previously validated to assess the binding of α-neurotoxins to taxa-specific orthosteric mimotopes [37, 46–48], to determine if primates might have evolved some resistance elements toward α-neurotoxins.

**Fig. 1.**
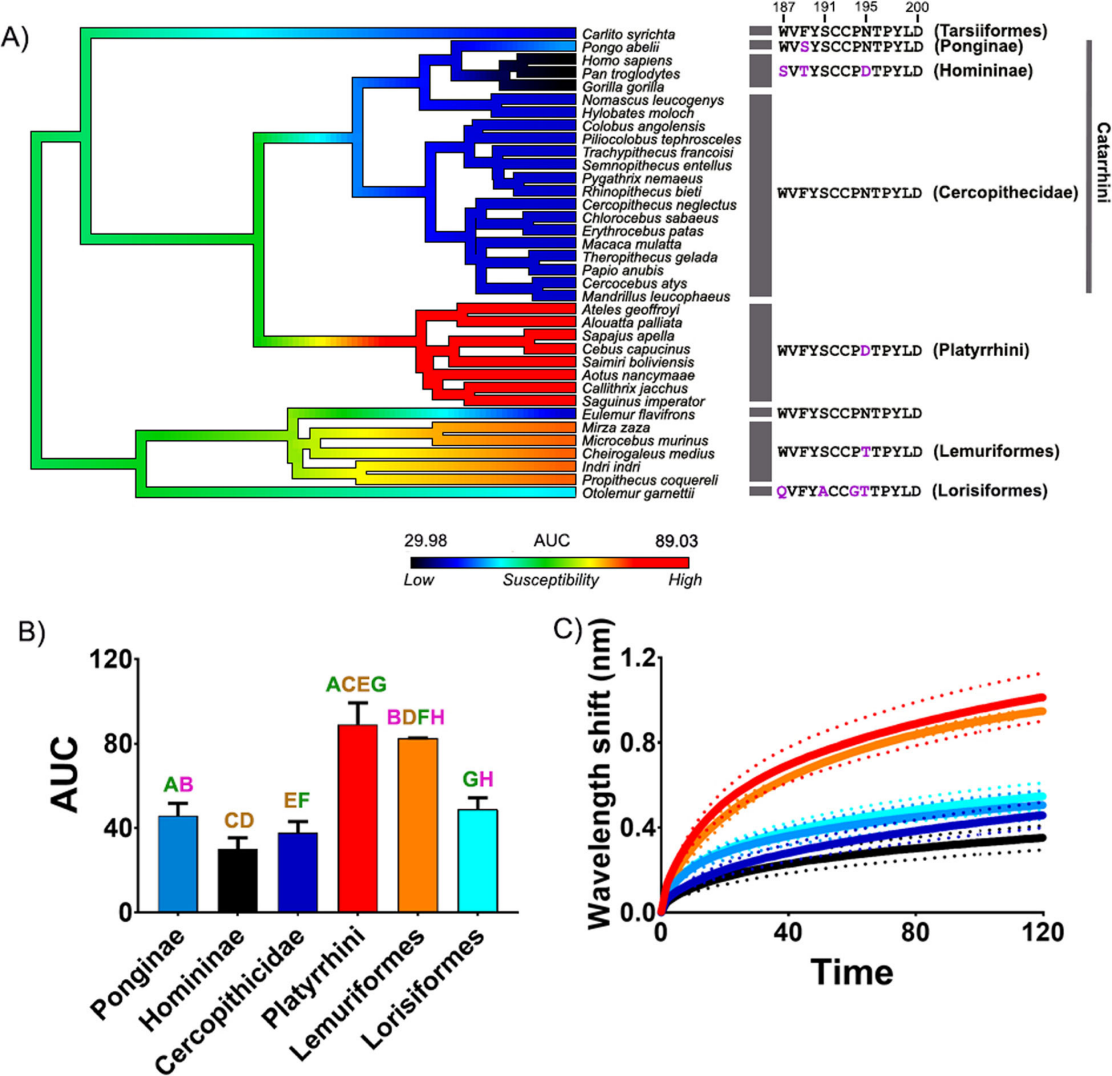
The effects of venom from the representative African cobra *Naja mossambica* against the nAChR orthosteric site mimotopes from seven clades of primates (see Additional file 1 for other African cobra species with congruent results). **A** Ancestral state reconstruction of the area under the curve (AUC) values of the binding of *N. mossambica* against the primate mimotopes. **B** Bar graphs represent the mean AUC values of the adjacent curve graphs. Statistical significance is indicated by matching letters with the colors of letter indicating the level of significance; brown *p*< 0.001, green *p*< 0.01, pink *p*< 0.05. **C** Curve graphs show the mean wavelength shift (nm) in light with binding of venoms over a 120-s association phase. The venom was tested in triplicate (*n*=3). Error bars on all graphs represent the SEM. AUC values were statistically analyzed using a one-way ANOVA with a Tukey’s comparisons multiple comparisons test comparing to the native mimotope. All raw data and statistical analyses outputs can be found in Additional file 2

**Fig. 2.**
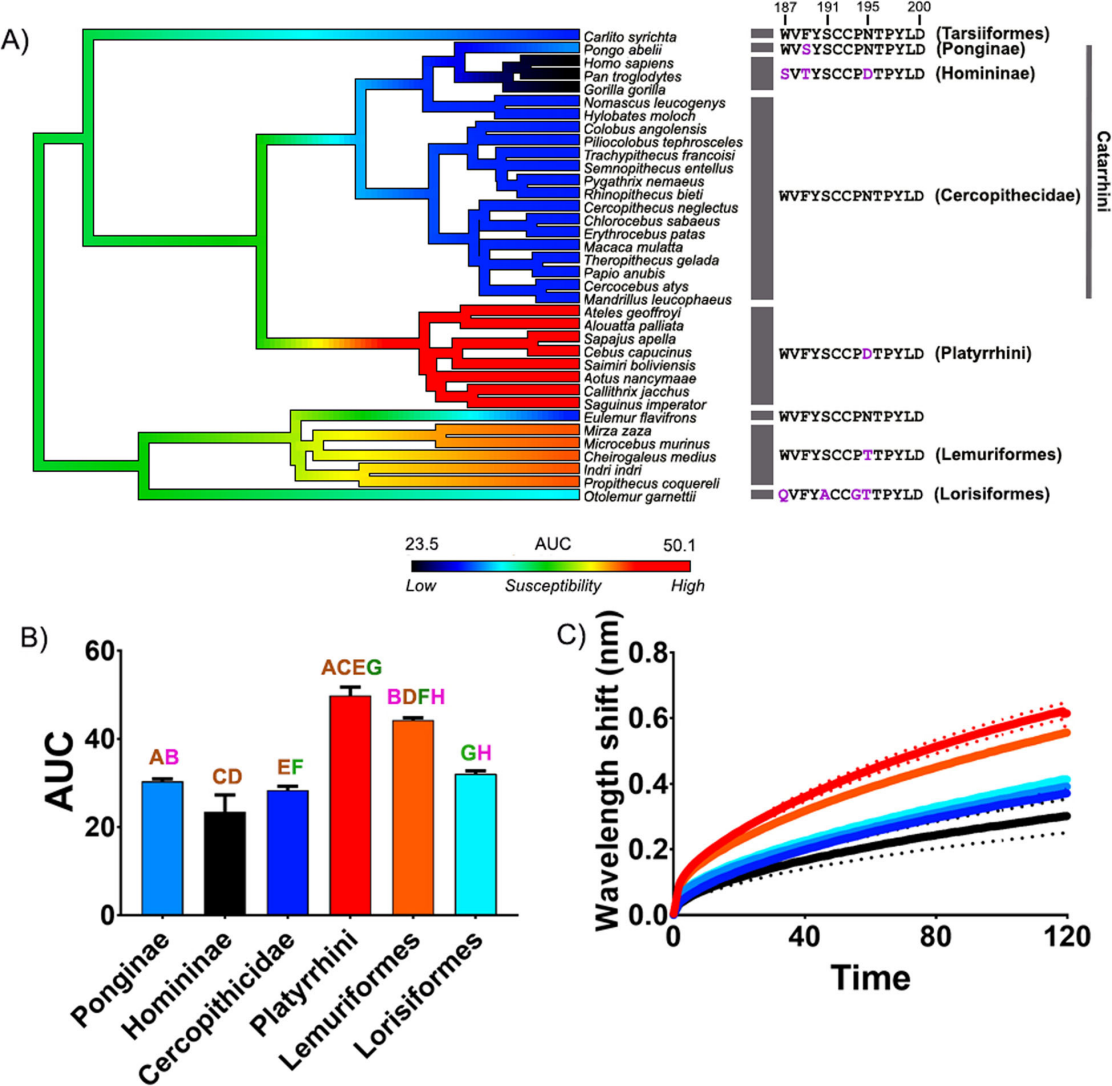
The effects of venom from the Asian cobra *Naja siamensis* against the nAChR orthosteric site mimotopes from seven clades of primates (see additional file 1 for other Asian cobra species with congruent results). **A** Ancestral state reconstruction of the area under the curve (AUC) values of the binding of *N. siamensis* against the primate mimotopes. **B** Bar graphs represent the mean AUC values of the adjacent curve graphs. Statistical significance is indicated by matching letters with the colors of letter indicating the level of significance; brown *p*< 0.001, green *p*< 0.01, pink *p*< 0.05. **C** Curve graphs show the mean wavelength shift (nm) in light with binding of venoms over a 120-s association phase. The venom was tested in triplicate (*n*=3). Error bars on all graphs represent the SEM. AUC values were statistically analyzed using a one-way ANOVA with a Tukey’s comparisons multiple comparisons test comparing to the native mimotope. All raw data and statistical analyses outputs can be found in Additional file 2

The results indicate a consistent binding pattern across all cobra venoms tested toward the primate lineage mimotopes (Figs. 1 and 2; Additional file 1: Figs S1-S3). The non-spitting species seem to have a much lower binding overall than the spitting, which would suggest lower proportions of orthosteric-site binding α-neurotoxic 3FTxs within their venom [47]. The difference observed in binding of the crude venoms is a consequence of the toxin interactions with the different biochemical properties of each amino acid present within the sequences [39, 49–51]. The binding patterns across all venoms tested showed a significantly increased resistance toward α-neurotoxins across the Homininae (Figs. 1 and 2; Additional file 1: Figs S1-S3). These data additionally support previous observations of weakly binding α-neurotoxins toward human nAChRs [37–40]. Further, the venoms also bound relatively weakly to the Cercopithecidae and Ponginae mimotopes (Figs. 1 and 2; Additional file 1: Figs S1-S3). The three clades, Cercopithecidae, Homininae, and Ponginae, occur in Africa and Asia, coinciding with the geographical distribution of cobras. The representatives of Lorisiformes and Tarsiiformes also have relatively low susceptibility, and both of these clades also occupy Africa and/or Asia. Conversely, the Lemuriformes (with the exception of *Eulemur flavifrons*) and Platyrrhini were the most susceptible to binding by the venoms (Figs. 1 and 2; Additional file 1: Figs S1-S3). Both of these clades are geographically separated from the African and Asian primate clades and also from diurnal elapids.

**Fig. 3.**
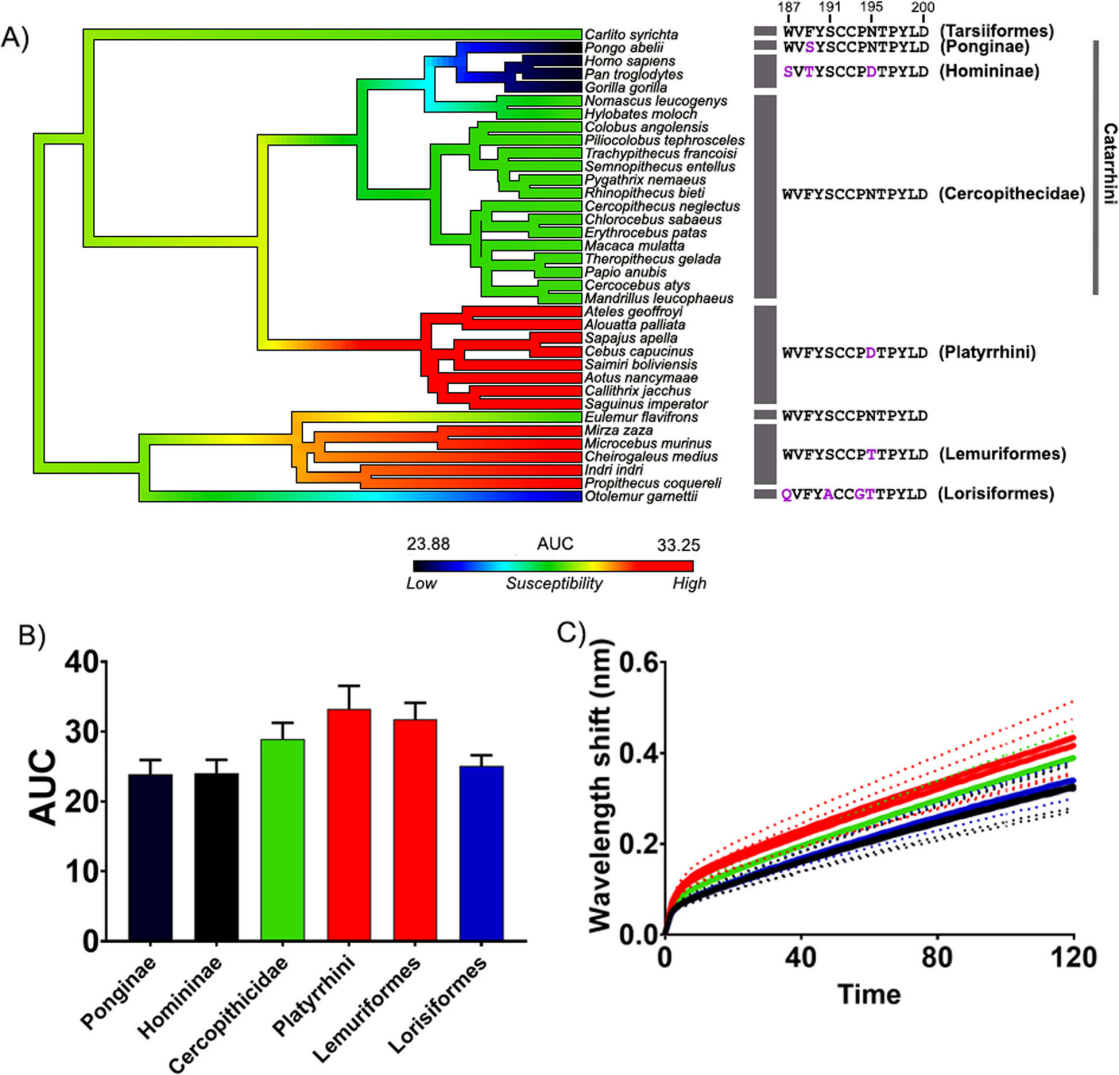
The effects of venom from the American coral snake *Micrurus browni* against the nAChR orthosteric site mimotopes from seven clades of primates. **A** Ancestral state reconstruction of the area under the curve (AUC) values of the binding of *M. browni* against the primate mimotopes. **B** Bar graphs represent the mean AUC values of the adjacent curve graphs. **C** Curve graphs show the mean wavelength shift (nm) in light with binding of venoms over a 120-s association phase. The venom was tested in triplicate (*n*=3). Error bars on all graphs represent the SEM. No statistical significance was detected using a one-way ANOVA. All raw data and statistical analyses outputs can be found in Additional file 2

**Fig. 4.**
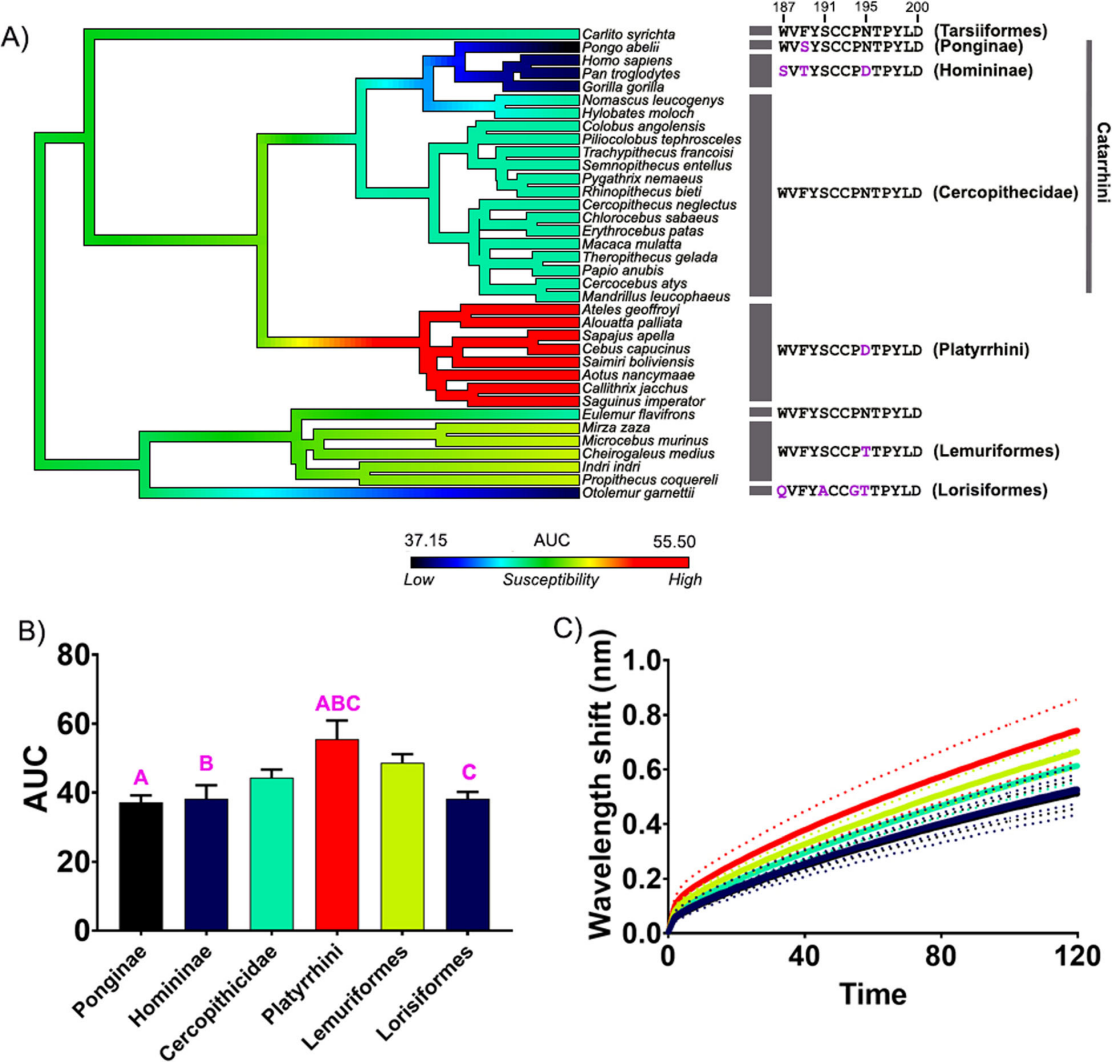
The effects of venom from the American coral snake *Micrurus corallinus* against the nAChR orthosteric site mimotopes from seven clades of primates. **A** Ancestral state reconstruction of the area under the curve (AUC) values of the binding of *M. corallinus* against the primate mimotopes. **B** Bar graphs represent the mean AUC values of the adjacent curve graphs. **C** Curve graphs show the mean wavelength shift (nm) in light with binding of venoms over a 120-s association phase. The venom was tested in triplicate (*n*=3). Error bars on all graphs represent the SEM. AUC values were statistically analyzed using a one-way ANOVA with a Tukey’s comparisons multiple comparisons test comparing to the native mimotope. A statistical significance is indicated by matching letters with the colors of letter indicating the level of significance; pink *p*< 0.01. All raw data and statistical analyses outputs can be found in Additional file 2

To further support the hypothesis that primates have evolved adaptations to reduce mortality from cobra encounters in Africa and Asia, we tested the binding of venoms from two species of American coral snakes (*Micrurus browni* and *M. corallinus*). The data show that there is no significance in binding between all primate groups of *M. browni* and a very small difference in binding between the clades of *M. corallinus* (Figs. 3 and 4) in comparison to that of cobras (Figs. 1 and 2; Additional file 1: Figs S1-S3). These data suggest that since there is a greater disparity between binding of both Platyrrhini and Lemuriformes for cobras but very little or no significant binding between these clades for coral snakes*,* then the strong selection pressure for the resistance across the African and Asian primates is likely from cobras.

**Fig. 5.**
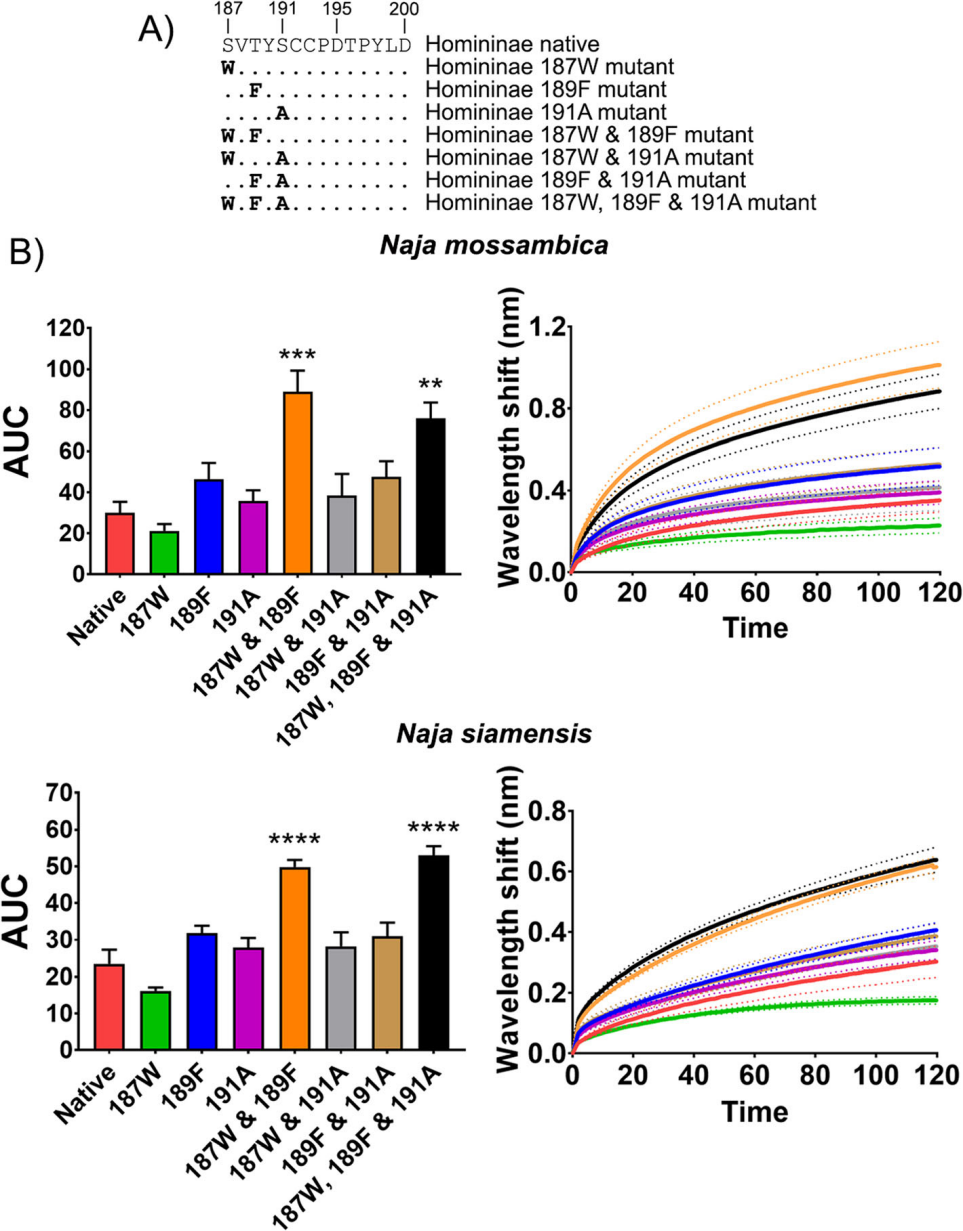
The effects of venom from a representative African cobra species (*Naja mossambica*) and a representative Asian cobra species (*N. siamensis*) against native and mutant Homininae mimotope sequences. **A** Amino acid sequences of native and mutant mimotopes. **B** Bar graphs represent the mean area under the curve (AUC) values of the adjacent curve graphs. Curve graphs show the mean wavelength (nm) shift in light with increased binding of venoms over a 120-s association phase. Each venom was tested in triplicate (*n*=3). Error bars on all graphs represent the SEM. AUC values were statistically analyzed using a one-way ANOVA with a Dunnett’s multiple comparisons post hoc test comparing to the native mimotope. A statistical significance is annotated above bars by ** (*p*< 0.01), *** (*p*< 0.001), or **** (*p*< 0.0001). All raw data and statistical analyses outputs can be found in Additional file 2

In order to ascertain the structure-activity relationships leading to hominines having the most reduced level of sensitivity to α-neurotoxins, we constructed mimotope mutants to elucidate key amino acid positions in the native sequence that are responsible for resistance. The bindings of representative African and Asian cobra venoms were tested against a series of orthosteric loop C site mutants with amino acid substitutions of the native hominin sequences. Each amino acid substituted was for that of the ancestral amino acid (Fig. 5). The results indicate that the amino acid positions 187 and 189 together seem to conform the bulk of the increased resistance since there was a significant increase in binding when these positions were substituted for the ancestral versions. These results were consistent for the representative African and Asian cobra venoms (Fig. 5). The amino acid substitutions within the Homininae sequence are biochemically polar, while the ancestral substitutions are non-polar. These polar amino acids likely provide enough of an electrostatic static repulsion in some instances to reduce the effective binding of the charged surface of the 3FTxs within the venoms. These results are also consistent with the previously untested hypothesis that lower binding of cobra venoms to humans is likely due to combinational substitutions at the aromatic subsite (positions 187 and 189) [39]. Further, there was an increase in susceptibility with all three amino acids at positions 187, 189, and 191 removed. All three of these positions are key binding sites for α-neurotoxins [28, 39, 50, 51], further confirming the importance of these positions in the binding of these toxin types to nAChRs.

## Discussion

All these data combined suggest that there was a strong selection pressure that has led to African and Asian primates becoming less susceptible toward cobra α-neurotoxin that bind to the orthosteric site, while primates living in other geographical areas were not subjected to these same selection pressures. The emerging patterns are consistent with the SDT [1] in that the primate clades that evolved in Africa and/or remained sympatric to venomous snakes show mechanisms to cope with certain selective pressures. Catarrhini, Lorisiformes, and Tarsiiformes have evolved an increased resistance toward α-neurotoxins whereas the Malagasy Lemuriformes have never co-existed with venomous snakes and the Platyrrhini occupy Central and South America where the elapids are small, crepuscular/nocturnal and fossorial, and thus pose little threat. The data further support the hypothesis that cobras and early hominins have been entwined in a coevolutionary arms-race resulting in the evolution of specific traits for both clades [15].

From an evolutionary perspective, it would seem that increased resistance convergently evolved on three occasions: once within Tarsiiformes (*Carlito syrichta*), once within Catarrhini (with resistance further amplified within Homininae) and once within Lorisiformes (*Otolemur garnettii*) (Figs. 1 and 2; Additional file 1: Figs S1-S3). Tarsiiformes and Lorisiformes likely evolved an increased resistance since they are small and prospective prey and in some cases predators to venomous snakes, whereas the larger Afro-Asian primates likely evolved the increased resistance due to an increase in terrestriality thus potentiating the number of possible encounters leading to defensive bites. Lemuriformes and Platyrrhini were never in contact with large venomous elapids as previously mentioned, and thus have never had a selection pressure to evolve any form of resistance. In the case of *E. flavifrons*, it is uncertain why there seems to be an increased resistance for this species while the other Lemuriformes did not evolve any since there is a lack of selection pressure. Conspicuously some dietary items of *Eulemur*, such as mushrooms and millipedes, contain high levels of neurotoxins [52]. Future work should investigate if the toxins from these sources target nAChRs (e.g., high concentrations of nicotine) and provide enough of a selection to maintain the lower susceptibility toward nAChR targeting toxins. There is likely an evolutionary trade-off disadvantage to fitness with evolving specific residue changes that lowers α-neurotoxin binding such as a reduced binding of the endogenous ACh [53]; thus, there must be an evolutionary selective advantage for *Eulemur* to have evolved this when the rest of the Lemuriformes did not.

Primates are largely an arboreal order, and the period during which spitting in cobras evolved occurred during a major climatic shift linked to a reduction in continuous forests [17]. During this time, diurnal primates would have needed to descend to the ground, and a number of species adapted to be fully terrestrial, increasing their chances of encountering and being defensively bitten by venomous elapid snakes. It should be noted that Ponginae are slightly less resistant than the Homininae clade, and this is possibly due to them being more arboreal than Homininae, reinforcing the importance of terrestriality in being exposed to elapids. Considering that snakes are can be prey items for some primates, and venomous snakes are a danger if startled and defensively bite, it is argued that snakes placed a tremendous selection pressure on anthropoid primates, to visually detect and avoid venomous snakes [1, 2].

One aspect of the SDT proposes that nocturnal strepsirrhines in Africa and Asia should have venom resistance since they were unable to expand their visual systems to the same extent as diurnal primates [2], which our data supports. However, this hypothesis went one step further suggesting that they would also have a greater venom resistance than other primates, which according to our data is not quite the case, but not entirely far from our observations either. Indeed, Lorisiformes and Tarsiiformes have relatively low susceptibility, with both of these clades also occupying Africa and/or Asia. They both also have particularly high orbital convergence, which has been associated with their coevolution with snakes [1]. Both of these clades consist of relatively small primates that are potential prey items for large venomous snakes. However, within the Lorisidae, the Sunda slow loris (*Nycticebus coucang*) contains a different type of resistance, that of the N-glycosylation motif (Table S1), which has been associated with extremely effective α-neurotoxin resistance [54–57]. This trait might have evolved due to a different selection pressure than that of *Otolemur garnettii*, since slow lorises have been documented eating venomous snakes. Slow lorises are also venomous themselves [58, 59], but given the extreme cytotoxicity of their venom and lack of neurotoxic symptoms, the N-glycosylation motif is unlikely to have evolved for autoresistance.

The greater increase in resistance within hominins coincides not only with obligate bipedalism and terrestriality, but also with the reduction of continuous forests requiring their movements into more open habitats. Foraging and traveling hominins were likely to come into contact much more often with terrestrial, diurnally active neurotoxic elapid snakes such as cobras, which would defend themselves against perceived threats using their venom [22]. The likely increased resistance toward α-neurotoxins from cobras might have driven cobras to develop a different kind of defense against hominins in the form of cytotoxins and spitting [15, 24]. An evolved form of partial resistance meant that cobra venom was not an effective method to reduce hominin interactions thus cytotoxins and spitting might have evolved in response as a deterrent. It has been proposed that spitting cobra clades evolved in response to hominines, and this might explain the difference we observed in binding between spitting and non-spitting cobras. A lower orthosteric site binding of α-neurotoxins in the venom of non-spitting than spitting cobras (likely due to the proportion of α-neurotoxins specific for this site within the venom) might be that the increased resistance across African and Asian primates has meant that spitters have had to evolve a high proportion of α-neurotoxins to overcome this increased resistance. Yet this proposed hypothesis needs careful additional consideration and investigation.

Further, since we have highlighted the selective coevolutionary pressures that might have come through hominin-snake interactions, it is entirely plausible that α-neurotoxin susceptibility differs between populations within humans. For example, populations that are highly prone to venomous snake conflicts and high rates of envenomations, such as Sub-Saharan Africa or rural India, may have a greater level of partial resistance than populations that never encounter wild elapids, such as within Europe. Though hypothetical, further research into variations of the orthosteric site sequences between different human populations could allow us to understand the effects of envenomations between different populations and aid our understanding of the snakebite crisis.

Thus, given that commonly encountered neurotoxic snakes exert a strong selection pressure in their defensive envenomations, why does there only seem to be an increased resistance rather than full resistance? Firstly, our aforementioned proposition that there might be an evolutionary trade-off disadvantage to evolving full resistance at the nAChR orthosteric site [53, 60] might mean that evolving partial resistance could allow for a balance between reduced α-neurotoxin susceptibility and a somewhat efficient ACh binding. This is supported by previous work revealing that increased resistance toward the nAChR agonist epibatidine decreased ACh sensitivity to the site [53]. Further, some vipers that are prey to α-neurotoxic snakes often display resistance, but this resistance is secondarily lost in populations which have dispersed into geographic areas devoid of neurotoxic snakes [57]. This differential resistance between sympatric and allopatric populations of venomous snakes and prey is also paralleled in ground squirrels and their rattlesnake predators, albeit a different mechanism of resistance [31]. Secondly, it might also be that partial resistance allows for a greater chance of survival against low levels of α-neurotoxins, since venomous snakes can control their injection volumes, often producing lower venom volumes during defensive bites to conserve the energetically costly venom for prey items rather than wasting it on a non-prey item [61–63]. Further to this point, human envenomations do not always result in death (even without antivenom intervention), thus partial resistance could also provide enough of an advantage to aid in survival in enough envenomation instances that the reduced susceptibility trait is effective enough to be maintained within a population. Thirdly, since primates are largely social animals [64, 65], it is possible that increased resistance might have been successful enough for kin selection [66–68], to maintain the partial resistance trait reciprocally. Having an increased resistance would allow the envenomed individual to fight against the snake for longer than more susceptible primates and also warn other members of the group that a venomous snake is present. Warning calls toward snakes occur within both nocturnal and diurnal primate groups [69, 70], thus being able to marginally extend an individual’s survival time to warn the group, allowing the safety of the relatives and, ultimately, their successful reproduction. This kin selection hypothesis certainly has its merits, while also raising more questions in terms of exactly how kin selection would be favored in light of partial resistance.

Although we have provided potential hypotheses for the coevolution of increased resistance within hominins that is well supported by data in this study and previous work, a competing hypothesis is that the resistance is due to simple genetic drift rather than being the result of evolutionary selection pressures. Under the evolutionary drift hypothesis, early hominins and primates in general tended to occur in small groups where genetic drift is most effective, thus the trait may have been maintained through this scenario. However, given the consilience of evidence suggesting a deeper coevolutionary history between snakes and primates, we think that our aforementioned hypotheses might hold a greater standing than something as innocuous as the genetic drift hypothesis. This thus provides a foundation upon which to conduct additional research to further strengthen and explore this fascinating evolutionary hypothesis.

## Conclusion

We have revealed an increased resistance across Afro-Asian primates toward snake venom α-neurotoxins that target nicotinic acetylcholine receptor orthosteric sites that seems to have convergently evolved on three occasions. There was a further amplification of resistance within the Homininae (chimpanzee, gorilla, human clade) when tested across African and Asian spitting and non-spitting cobras. This finding is consistent with clinical symptoms of neurotoxicity being reduced for α-neurotoxins that bind to the orthosteric site, relative to the natural prey types. Conversely, the Lemuriformes and Platyrrhini have the highest susceptibility of primates tested toward α-neurotoxic venoms. This coincides with the evolutionary biogeography of these lineages in Madagascar and South/Central America respectively, evolving in the absence of large neurotoxic snake species. Our data also further support one of the hypotheses proposed by the Snake Detection Theory, whereby venomous snakes and primates share a history of coevolution through their continuous co-existence across Africa and Asia [1], as evidenced by the venom of two species of coral snakes tested here show little or no disparity between primate group mimotope binding. These data further support the hypothesis that cobras and hominins have evolved certain traits through a coevolutionary arms race. The evidence in support of the coevolution of primates and snakes seems to be increasing, yet despite this, fundamental gaps in knowledge remain both regarding the ecology of snakes and primates and how their shared coevolutionary history might have brought about some of their most distinguishing traits. Future work should investigate why partial rather than full resistance has evolved, including our hypothesis of kin selection.

## Methods

### Venom collection and preparation

All venom work was undertaken under the auspices of UQ IBSC approval #IBC134BSBS2015. Venoms were pools of adult snakes (*N*=3) to minimize the effect of individual variation from the long-term cryogenic collection of the Venom Evolution Lab. All venom samples were lyophilized and reconstituted in double deionized water (ddH_2_O), and centrifuged (4 °C, 10 min at 14,000 relative centrifugal force (RCF)). The supernatant was made into a working stock (1 mg/mL) in 50% glycerol at − 20 °C. The concentrations of experimental stocks were determined using a NanoDrop 2000 UV-Vis Spectrophotometer (Thermo Fisher, Sydney, Australia) at an absorbance wavelength of 280 nm.

### Mimotope production and preparation

Following methods from a previously developed assay [47, 48], a 13–14 amino acid mimotope of the vertebrate α-1 nAChR orthosteric site was developed by GenicBio Ltd. (Shanghai, China) designed upon specification. Mimotopes from lineage representatives were as follows; *Homo sapiens* (G5E9G9), *Otolemur garnettii* (H0WHF2_OTOGA), *Pongo abelii* (H2P7W2_PONAB), lemur/mouse (P04756), Cercopithecidae/rodent (P25108), and Platyrrhini/*Nomascus leucogenys* (ENSNLET00000007512.3). The C–C of the native mimotope is replaced during peptide synthesis with S–S to avoid uncontrolled postsynthetic thiol oxidation. The C–C bond in the nAChR binding region does not participate directly in analyte-ligand binding [51, 71, 72], thus replacement to S–S is not expected to have any effect on the analyte-ligand complex formation. However, the presence of the C–C bridge is key in the conformation of the interaction site of whole receptors [73]. As such, we suggest direct comparisons of kinetics data, such as Ka or KD, between nAChR mimotopes and whole receptor testing should be avoided, or at least approached with caution. Mimotopes were further synthesized to a biotin linker bound to two aminohexanoic acid (Ahx) spacers, forming a 30-Å linker. Mimotope dried stocks were solubilized in 100% dimethyl sulfoxide (DMSO) and diluted in ddH_2_O at 1:10 dilution to obtain a stock concentration of 50 μg/mL. Stocks were stored at − 80 °C until required.

### Biolayer Interferometry (BLI)

Full details of the developed assay, including all methodology and data analysis, can be found in the validated protocol [48] and further data using this protocol [37, 47]. In brief, the BLI assay was performed on the Octet HTX system (ForteBio, Fremont, CA, USA). Venom samples were diluted 1:20, making a final concentration of 50 μg/mL per well. Mimotope aliquots were diluted 1:50, with a final concentration of 1 μg/mL per well. The assay running buffer was 1X DPBS with 0.1% BSA and 0.05% Tween-20. Preceding experimentation, Streptavidin biosensors were hydrated in the running buffer for 30–60 min, while on a shaker at 2.0 revolutions per minute (RPM). The dissociation of analytes occurred using a standard acidic glycine buffer solution (10 mM glycine (pH 1.5–1.7) in ddH_2_O). Raw data are provided in Additional file 1.

### Data processing and analysis

All data obtained from BLI on Octet HTX system (ForteBio) were processed in exact accordance to the validation of this assay [48]. The association step data were obtained and imported into Prism8.0 software (GraphPad Software Inc., La Jolla, CA, USA) where area under the curve (AUC) and one-way ANOVA with Tukey’s multiple comparisons analyses were conducted and graphs produced.

Phylogenetic trees were obtained from timetree.org. The obtained phylogenetic trees were then further analyzed in RStudio (R Core Team, 2015) for all comparative analyses using the Ape package [74]. Heat-mapping of AUC values over the phylogenetic trees was achieved using the contMap function of the R package phytools [75].

## Supplementary Information

**Additional file 1: Fig. S1.** The effects of venom from an additional African cobra species (*Naja nubiae*) against the nAChR orthosteric site mimotopes from seven clades of primates. **Fig. S2.** The effects of venom from an additional African cobra species (*Naja haje*) against the nAChR orthosteric site mimotopes from seven clades of primates. **Fig. S3.** The effects of venom from an additional Asian cobra species (*Naja kaouthia*) against the nAChR orthosteric site mimotopes from seven clades of primates. **Table S1.** Orthosteric site sequences for each species and public database accessions codes.

**Additional file 2:.** All the raw experimental data output and statistical analyses.

## Data Availability

All data generated or analyzed during this study are included in this published article and Additional files 1 and 2.

## Abbreviations

ACh: Acetylcholine
BLI: Biolayer interferometry
MA: Magna anum (one million years)
nAChR: Nicotinic acetylcholine receptor
SDT: Snake Detection Theory
3FTx: Three-finger toxin

## References

[1] Isbell LA (2006). Snakes as agents of evolutionary change in primate brains. J Human Evol.

[2] L. A. Isbell, The fruit, the tree, and the serpent (Harvard University Press, 2009), DOI: 10.2307/j.ctvjnrvj0.

[3] Öhman A, Mineka S (2003). The malicious serpent: snakes as a prototypical stimulus for an evolved module of fear. Curr Direct Psychol Sci.

[4] Soares SC, Lindström B, Esteves F, Öhman A (2014). The hidden snake in the grass: superior detection of snakes in challenging attentional conditions. PLoS one.

[5] Soares SC (2012). The lurking snake in the grass: interference of snake stimuli in visually taxing conditions. Evol Psychol.

[6] Le QV (2013). Pulvinar neurons reveal neurobiological evidence of past selection for rapid detection of snakes. Proceed Natl Acad Sci.

[7] Isbell LA, Etting SF. Scales drive detection, attention, and memory of snakes in wild vervet monkeys (*Chlorocebus pygerythrus*). Primates. 2017;58(1):121–9. 10.1007/s10329-016-0562-y. 10.1007/s10329-016-0562-y 27517268

[8] Yoder AD, Yang Z (2004). Divergence dates for Malagasy lemurs estimated from multiple gene loci: geological and evolutionary context. Molecular Ecology.

[9] Alencar LR (2016). Diversification in vipers: Phylogenetic relationships, time of divergence and shifts in speciation rates. Mol Phylogenet Evol.

[10] Lee MS, Sanders KL, King B, Palci A (2016). Diversification rates and phenotypic evolution in venomous snakes (Elapidae). R Soc Open Sci.

[11] Schrago CG, Russo CA (2003). Timing the origin of New World monkeys. Mol Biol Evol.

[12] Zamudio KR, Greene HW. Phylogeography of the bushmaster (*Lachesis muta*: Viperidae): implications for neotropical biogeography, systematics, and conservation. Biol J Linnean Soc. 1997;62(3):421–42. 10.1006/bijl.1997.0162.

[13] W. Wüster et al., Origins and evolution of the South American pitviper fauna: evidence from mitochondrial DNA sequence analysis. Biology of the Vipers, 111-128 (2002).

[14] Wüster W, Peppin L, Pook CE, Walker DE (2008). A nesting of vipers: phylogeny and historical biogeography of the Viperidae (Squamata: Serpentes). Mol Phylogenet Evol.

[15] Kazandjian TD, Petras D, Robinson SD, van Thiel J, Greene HW, Arbuckle K, Barlow A, Carter DA, Wouters RM, Whiteley G, Wagstaff SC, Arias AS, Albulescu LO, Plettenberg Laing A, Hall C, Heap A, Penrhyn-Lowe S, McCabe CV, Ainsworth S, da Silva RR, Dorrestein PC, Richardson MK, Gutiérrez JM, Calvete JJ, Harrison RA, Vetter I, Undheim EAB, Wüster W, Casewell NR (2021). Convergent evolution of pain-inducing defensive venom components in spitting cobras. Science.

[16] Pozzi L, Hodgson JA, Burrell AS, Sterner KN, Raaum RL, Disotell TR (2014). Primate phylogenetic relationships and divergence dates inferred from complete mitochondrial genomes. Molecular Phylogenetics and Evolution.

[17] Andrews P (2020). Last common ancestor of apes and humans: Morphology and environment. Folia Primatologica.

[18] M. O'Shea, The Book of Snakes: A life-size guide to six hundred species from around the world (University of Chicago Press, 2018), DOI: 10.7208/chicago/9780226459424.001.0001.

[19] McGrew WC (2015). Snakes as hazards: modelling risk by chasing chimpanzees. Primates.

[20] Carotenuto F, Tsikaridze N, Rook L, Lordkipanidze D, Longo L, Condemi S, Raia P (2016). Venturing out safely: the biogeography of Homo erectus dispersal out of Africa. Journal of Human Evolution.

[21] Greene HW (2017). Evolutionary scenarios and primate natural history. The American Naturalist.

[22] Headland TN, Greene HW (2011). Hunter–gatherers and other primates as prey, predators, and competitors of snakes. Proceedings of the National Academy of Sciences.

[23] Boinski S. Use of a club by a wild white-faced capuchin (*Cebus capucinus*) to attack a venomous snake (Bothrops asper). American Journal of Primatology. 1988;14(2):177–9. 10.1002/ajp.1350140208. 10.1002/ajp.1350140208 31973450

[24] Panagides N, Jackson T, Ikonomopoulou M, Arbuckle K, Pretzler R, Yang D, Ali S, Koludarov I, Dobson J, Sanker B, Asselin A, Santana R, Hendrikx I, van der Ploeg H, Tai-A-Pin J, van den Bergh R, Kerkkamp H, Vonk F, Naude A, Strydom M, Jacobsz L, Dunstan N, Jaeger M, Hodgson W, Miles J, Fry B (2017). How the cobra got its flesh-eating venom: Cytotoxicity as a defensive innovation and its co-evolution with hooding, aposematic marking, and spitting. Toxins.

[25] Drabeck DH, Dean AM, Jansa SA (2015). Why the honey badger don’t care: Convergent evolution of venom-targeted nicotinic acetylcholine receptors in mammals that survive venomous snake bites. Toxicon.

[26] Holding ML, Biardi JE, Gibbs HL (2016). Coevolution of venom function and venom resistance in a rattlesnake predator and its squirrel prey. Proceedings of the Royal Society of London B: Biological Sciences.

[27] Holding ML, Putman BJ, Kong LM, Smith JE, Clark RW (2020). Physiological stress integrates resistance to rattlesnake venom and the onset of risky foraging in California ground squirrels. Toxins.

[28] Kachalsky SG, Jensen BS, Barchan D, Fuchs S (1995). Two subsites in the binding domain of the acetylcholine receptor: an aromatic subsite and a proline subsite. Proceedings of the National Academy of Sciences.

[29] Drabeck DH, Rucavado A, Hingst-Zaher E, Cruz YP, Dean AM, Jansa SA (2020). Resistance of South American opossums to vWF-binding venom C-type lectins. Toxicon.

[30] S. A. Jansa, R. S. Voss, Adaptive evolution of the venom-targeted vWF protein in opossums that eat pitvipers. PLoS One 6 (2011). 10.1371/journal.pone.0020997 PMC3120824 21731638

[31] Coss RG, Poran NS, Gusé KL, Smith DG. Development of antisnake defenses in California ground squirrels (*Spermophilus beecheyi*): II. Microevolutionary effects of relaxed selection from rattlesnakes. Behaviour. 1993;124(1-2):137–62. 10.1163/156853993X00542.

[32] Gotti C, Clementi F (2004). Neuronal nicotinic receptors: from structure to pathology. Progress in Neurobiology.

[33] Fambrough DM (1979). Control of acetylcholine receptors in skeletal muscle. Physiological Reviews.

[34] Galzi J, Revah F, Bessis A, Changeux J (1991). Functional architecture of the nicotinic acetylcholine receptor: from electric organ to brain. Ann Rev Pharmaco Toxicol.

[35] Barber CM, Isbister GK, Hodgson WC (2013). Alpha neurotoxins. Toxicon.

[36] Nirthanan S, Gwee MC (2004). Three-finger α-neurotoxins and the nicotinic acetylcholine receptor, forty years on. J Pharmacol Sci.

[37] Harris RJ, Zdenek CN, Debono J, Harrich D, Fry BG (2020). Evolutionary interpretations of nicotinic acetylcholine receptor targeting venom effects by a clade of Asian Viperidae snakes. Neurotoxic Res.

[38] Ishikawa Y, Kano M, Tamiya N, Shimada Y (1985). Acetylcholine receptors of human skeletal muscle: A species difference detected by snake neurotoxins. Brain Research.

[39] Dellisanti C, Yao Y, Stroud JC, Wang Z-Z, Chen L (2007). Structural determinants for α-neurotoxin sensitivity in muscle nAChR and their implications for the gating mechanism. Channels.

[40] Silva A, Cristofori-Armstrong B, Rash LD, Hodgson WC, Isbister GK (2018). Defining the role of post-synaptic α-neurotoxins in paralysis due to snake envenoming in humans. Cellular and molecular life sciences.

[41] Fry BG, Lumsden NG, Wister W, Wickramaratna JC, Hodgson WC, Manjunatha Kini R (2003). Isolation of a neurotoxin (α-colubritoxin) from a nonvenomous colubrid: evidence for early origin of venom in snakes. J Mol Evol.

[42] Pawlak J, Mackessy SP, Fry BG, Bhatia M, Mourier G, Fruchart-Gaillard C, et al. Denmotoxin, a three-finger toxin from the colubrid snake *Boiga dendrophila* (Mangrove Catsnake) with bird-specific activity. Journal of Biological Chemistry. 2006;281(39):29030–41. 10.1074/jbc.M605850200. 10.1074/jbc.M605850200 16864572

[43] Pawlak J, Mackessy SP, Sixberry NM, Stura EA, le du MH, Ménez R, Foo CS, Ménez A, Nirthanan S, Kini RM (2009). Irditoxin, a novel covalently linked heterodimeric three-finger toxin with high taxon-specific neurotoxicity. The FASEB Journal.

[44] Heyborne WH, Mackessy SP. Identification and characterization of a taxon-specific three-finger toxin from the venom of the Green Vinesnake (*Oxybelis fulgidus*; family Colubridae). Biochimie. 2013;95(10):1923–32. 10.1016/j.biochi.2013.06.025. 10.1016/j.biochi.2013.06.025 23851011

[45] Mackessy SP, Sixberry NM, Heyborne WH, Fritts T. Venom of the Brown Treesnake, *Boiga irregularis*: ontogenetic shifts and taxa-specific toxicity. Toxicon. 2006;47(5):537–48. 10.1016/j.toxicon.2006.01.007. 10.1016/j.toxicon.2006.01.007 16545413

[46] Harris RJ, Youngman NJ, Zdenek CN, Huynh TM, Nouwens A, Hodgson WC, Harrich D, Dunstan N, Portes-Junior JA, Fry BG (2020). Assessing the binding of venoms from aquatic elapids to the nicotinic acetylcholine receptor orthosteric site of different prey models. International Journal of Molecular Sciences.

[47] Harris RJ, Zdenek CN, Harrich D, Frank N, Fry BG (2020). An appetite for destruction: Detecting prey-selective binding of α-neurotoxins in the venom of Afro-Asian elapids. Toxins.

[48] Zdenek CN, Harris RJ, Kuruppu S, Youngman NJ, Dobson JS, Debono J, Khan M, Smith I, Yarski M, Harrich D, Sweeney C, Dunstan N, Allen L, Fry BG (2019). A taxon-specific and high-throughput method for measuring ligand binding to nicotinic acetylcholine receptors. Toxins.

[49] Barchan D, Ovadia M, Kochva E, Fuchs S (1995). The binding site of the nicotinic acetylcholine receptor in animal species resistant to. alpha-bungarotoxin. Biochemistry.

[50] C. D. Dellisanti, Y. Yao, J. C. Stroud, Z.-Z. Wang, L. Chen, Crystal structure of the extracellular domain of nAChR α1 bound to α-bungarotoxin at 1.94 Å resolution. Nature neuroscience 10, 953-962 (2007). 10.1038/nn1942 17643119

[51] S. Tzartos, M. S. Remoundos, Fine localization of the major alpha-bungarotoxin binding site to residues alpha 189-195 of the Torpedo acetylcholine receptor. Residues 189, 190, and 195 are indispensable for binding. Journal of Biological Chemistry 265, 21462-21467 (1990). 2254308

[52] Peckre LR, Defolie C, Kappeler PM, Fichtel C (2018). Potential self-medication using millipede secretions in red-fronted lemurs: combining anointment and ingestion for a joint action against gastrointestinal parasites?. Primates.

[53] Tarvin RD, Borghese CM, Sachs W, Santos JC, Lu Y, O’Connell LA, Cannatella DC, Harris RA, Zakon HH (2017). Interacting amino acid replacements allow poison frogs to evolve epibatidine resistance. Science.

[54] Barchan D, Kachalsky S, Neumann D, Vogel Z, Ovadia M, Kochva E, Fuchs S (1992). How the mongoose can fight the snake: the binding site of the mongoose acetylcholine receptor. Proceed Natl Acad Sci.

[55] Takacs Z, Wilhelmsen KC, Sorota S. Snake α-neurotoxin binding site on the Egyptian cobra (*Naja haje*) nicotinic acetylcholine receptor is conserved. Mol Biol Evol. 2001;18(9):1800–9. 10.1093/oxfordjournals.molbev.a003967. 10.1093/oxfordjournals.molbev.a003967 11504859

[56] Takacs Z, Wilhelmsen KC, Sorota S. Cobra (Naja spp.) nicotinic acetylcholine receptor exhibits resistance to erabu sea snake (*Laticauda semifasciata*) short-chain α-neurotoxin. J Mol Evol. 2004;58:516–26. 10.1007/s00239-003-2573-8 15170255

[57] Khan MA, Dashevsky D, Kerkkamp H, Kordiš D, de Bakker MAG, Wouters R, van Thiel J, op den Brouw B, Vonk FJ, Kini RM, Nazir J, Fry BG, Richardson MK (2020). Widespread evolution of molecular resistance to snake venom α-neurotoxins in vertebrates. Toxins.

[58] Madani G, Nekaris KA-I. Anaphylactic shock following the bite of a wild Kayan slow loris (*Nycticebus kayan*): implications for slow loris conservation. J VenomAnim Toxins Trop Dis. 2014;20(1):1–5. 10.1186/1678-9199-20-43. 10.1186/1678-9199-20-43 PMC4192448 25309586

[59] Nekaris KA-I, Moore RS, Rode EJ, Fry BG (2013). Mad, bad and dangerous to know: the biochemistry, ecology and evolution of slow loris venom. J Venom Anim Toxins Trop Dis.

[60] Harris RJ, Fry BG (2021). Electrostatic resistance to alpha-neurotoxins conferred by charge reversal mutations in nicotinic acetylcholine receptors. Proceedings of the Royal Society B.

[61] Hayes WK, Herbert SS, Harrison JR, Wiley KL (2008). Spitting versus biting: differential venom gland contraction regulates venom expenditure in the black-necked spitting cobra, Naja nigricollis nigricollis. J Herpetol.

[62] Young BA, Zahn K (2001). Venom flow in rattlesnakes: mechanics and metering. J Exper Biol.

[63] Morgenstern D, King GF (2013). The venom optimization hypothesis revisited. Toxicon.

[64] J. B. Silk, P. M. Kappeler, Sociality in primates. Comparative Social Evolution, 253-283 (2017).

[65] J. Terborgh, C. Janson, The socioecology of primate groups. Annual Review of Ecology and Systematics, 111-136 (1986).

[66] Kurland JA (1980). Kin selection theory: a review and selective bibliography. Ethol Sociobiol.

[67] J. B. Silk, "Practicing Hamilton’s rule: kin selection in primate groups" in Cooperation in primates and humans. (Springer, 2006), pp. 25-46.

[68] Silk JB (2002). Kin selection in primate groups. Int J Primatol.

[69] Ramakrishnan U, Coss RG, Schank J, Dharawat A, Kim S. Snake species discrimination by wild bonnet macaques (*Macaca radiata*). Ethology. 2005;111(4):337–56. 10.1111/j.1439-0310.2004.01063.x.

[70] Seyfarth RM, Cheney DL, Marler P (1980). Monkey responses to three different alarm calls: evidence of predator classification and semantic communication. Science.

[71] McLane KE, Wu X, Conti-Tronconi BM. An α-bungarotoxin-binding sequence on the *Torpedo* nicotinic acetylcholine receptor α-subunit: conservative amino acid substitutions reveal side-chain specific interactions. Biochemistry. 1994;33(9):2576–85. 10.1021/bi00175a029. 10.1021/bi00175a029 8117719

[72] K. E. McLane, X. Wu, B. Diethelm, B. M. Conti-Tronconi, Structural determinants of α-bungarotoxin binding to the sequence segment 181-200 of the muscle nicotinic acetylcholine receptor. α-subunit: effects of cysteine/cystine modification and species-specific amino acid substitutions. Biochemistry 30, 4925-4934 (1991). 10.1021/bi00234a013 2036361

[73] Testai FD, Venera GD, Peña C, de Jiménez Bonino MJB (2000). Histidine 186 of the nicotinic acetylcholine receptor α subunit requires the presence of the 192–193 disulfide bridge to interact with α-bungarotoxin. Neurochem Int.

[74] Paradis E, Claude J, Strimmer K (2004). APE: analyses of phylogenetics and evolution in R language. Bioinformatics.

[75] L. J. Revell, phytools: an R package for phylogenetic comparative biology (and other things). Methods in Ecology and Evolution 3, 217-223 (2012).

