# Supplementary material for: Monkeying around with venom: an increased resistance to α-neurotoxins supports an evolutionary arms race between Afro-Asian primates and sympatric cobras
Source: BMC Biol. 2021 Nov 25;19:253. doi: 10.1186/s12915-021-01195-x (PMC8613972; doi:10.1186/s12915-021-01195-x)
Supplement: Supplementary file 1 — Additional file 1: Fig. S1. The effects of venom from an additional African cobra species (Naja nubiae) against the nAChR orthosteric site mimotopes from seven clades of primates. Fig. S2. The effects of venom from an additional African cobra species (Naja haje) against the nAChR orthosteric site mimotopes from seven clades of primates. Fig. S3. The effects of venom from an additional Asian cobra species (Naja kaouthia) against the nAChR orthosteric site mimotopes from seven clades of primates. Table S1. Orthosteric site sequences for each species and public database accessions codes. [file 12915_2021_1195_MOESM1_ESM.docx]

**ADDITIONAL FILE 1**


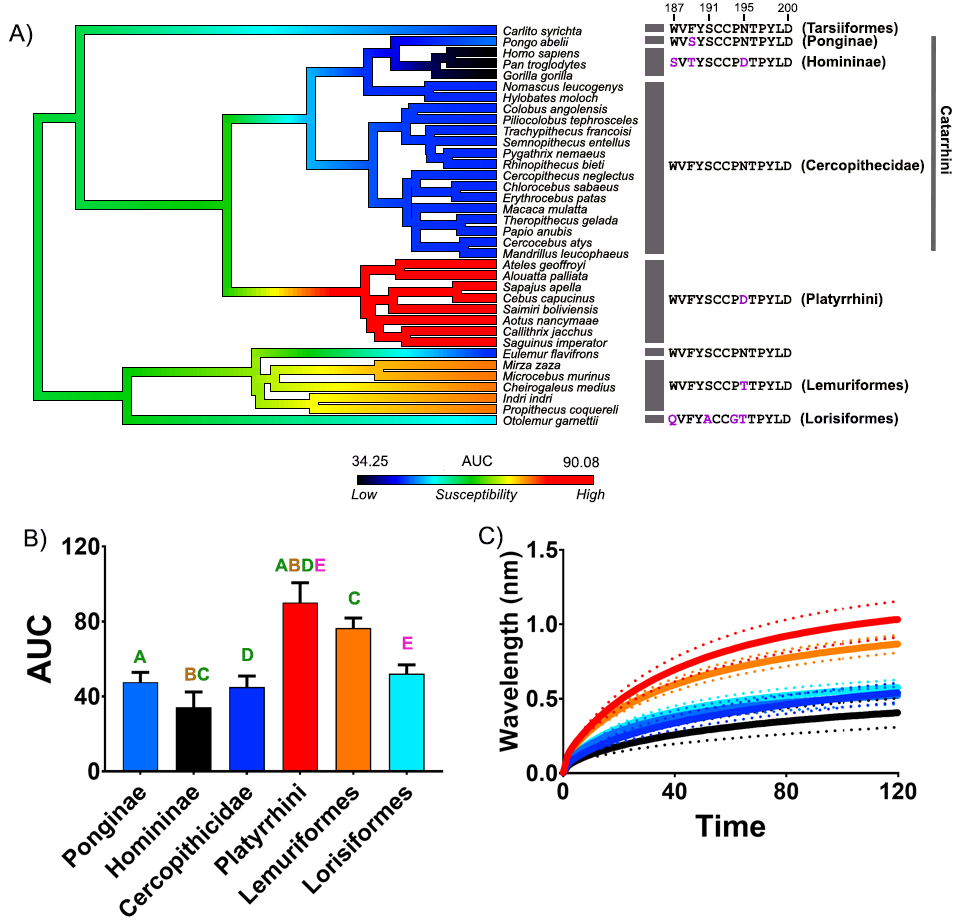


**Fig. S1.** **The effects of venom from an additional African cobra species (*Naja nubiae*) against the nAChR orthosteric site mimotopes from seven clades of primates.** A) Ancestral state reconstruction of the area under the curve (AUC) values of the binding of *N. nubiae* against the primate mimotopes. B) Bar graphs represent the mean AUC values of the adjacent curve graphs. C) Curve graphs show the mean wavelength shift (nm) in light with binding of venoms over a 120 second association phase. The venom was tested in triplicate (n=3). Error bars on all graphs represent the SEM. AUC values were statistically analysed using a one-way ANOVA with a Tukey’s comparisons multiple comparisons test comparing to the native mimotope. A statistical significance is indicated by matching letters with the colours of letter indicating the level of significance; brown p<0.001, green p<0.01, pink p<0.05. All raw data and statistical analyses outputs can be found in Additional file 2.


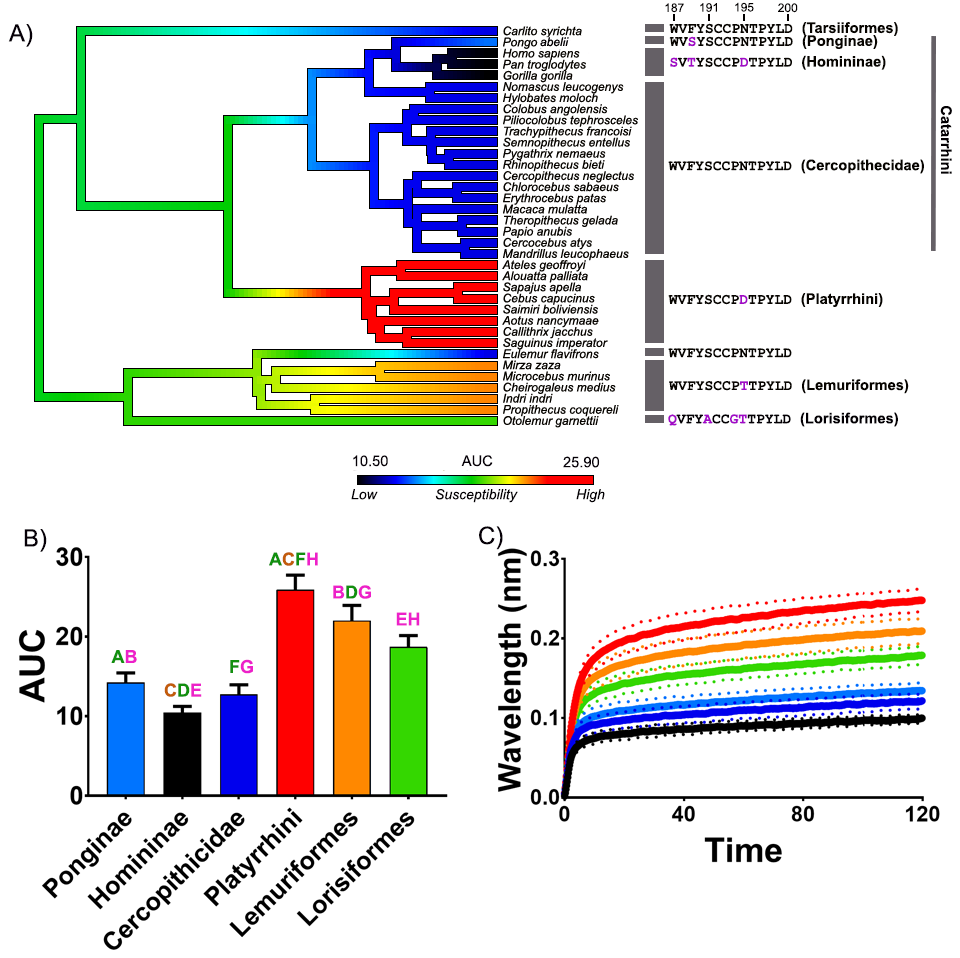
 **Fig. S2.** **The effects of venom from an additional African cobra species (*Naja haje*) against the nAChR orthosteric site mimotopes from seven clades of primates.** A) Ancestral state reconstruction of the area under the curve (AUC) values of the binding of *N. haje* against the primate mimotopes. B) Bar graphs represent the mean AUC values of the adjacent curve graphs. C) Curve graphs show the mean wavelength shift (nm) in light with binding of venoms over a 120 second association phase. The venom was tested in triplicate (n=3). Error bars on all graphs represent the SEM. AUC values were statistically analysed using a one-way ANOVA with a Tukey’s comparisons multiple comparisons test comparing to the native mimotope. A statistical significance is indicated by matching letters with the colours of letter indicating the level of significance; brown p<0.0001, green p<0.001, pink p<0.05. All raw data and statistical analyses outputs can be found in Additional file 2.


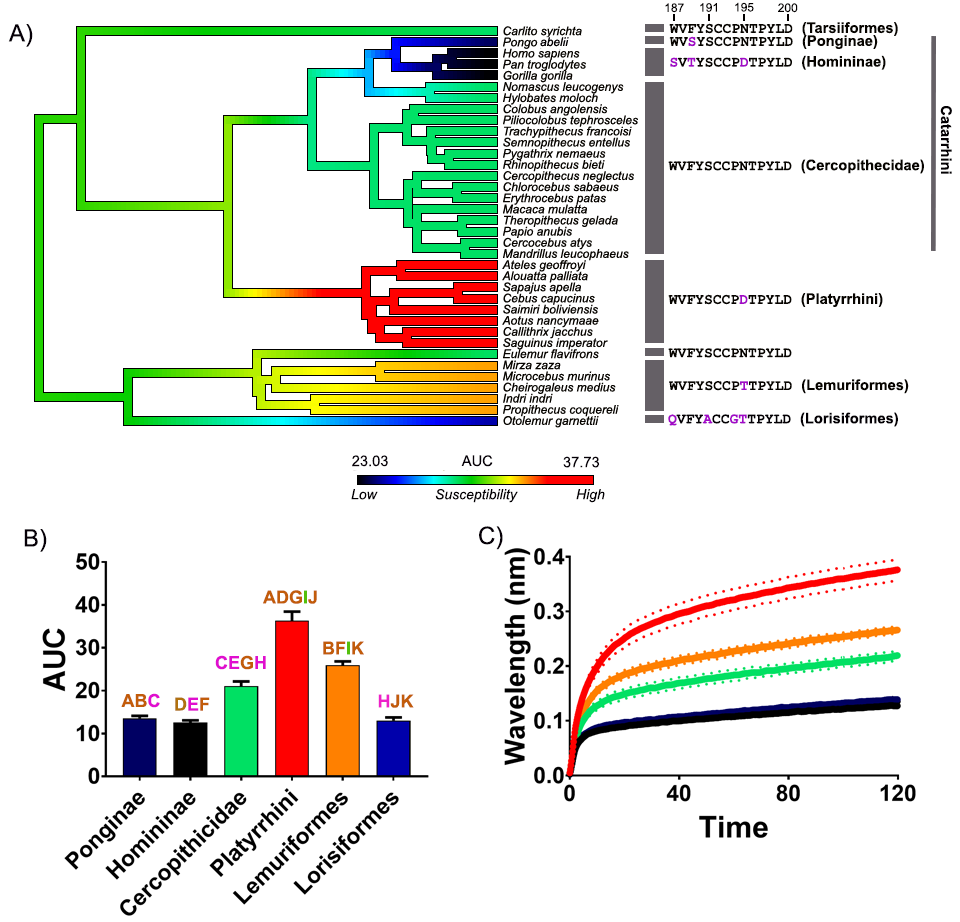


**Fig. S3.** **The effects of venom from an additional Asian cobra species (*Naja kaouthia*) against the nAChR orthosteric site mimotopes from seven clades of primates.** A) Ancestral state reconstruction of the area under the curve (AUC) values of the binding of *N. kaouthia* against the primate mimotopes. B) Bar graphs represent the mean AUC values of the adjacent curve graphs. C) Curve graphs show the mean wavelength shift (nm) in light with binding of venoms over a 120 second association phase. The venom was tested in triplicate (n=3). Error bars on all graphs represent the SEM. AUC values were statistically analysed using a one-way ANOVA with a Tukey’s comparisons multiple comparisons test comparing to the native mimotope. A statistical significance is indicated by matching letters with the colours of letter indicating the level of significance; brown p<0.0001, green p<0.001, pink p<0.05. All raw data and statistical analyses outputs can be found in Additional file 2.

**Table S1.** Orthosteric site sequences for each species and public database accessions codes**.**

| **Species** | **Common name** | **Sequence** | **Lineage** | **Accession code** |
| --- | --- | --- | --- | --- |
| *Otolemur garnettii* | Small-eared galago | QVFYACCGTTPYLD | Lorisiformes | H0WHF2 |
| *Propithecus coquereli* | Coquerel's sifaka | WVFYSCCPTTPYLD | Lemuriformes | A0A2K6FUM4 |
| *Indri Indri* | Indri | WVFYSCCPTTPYLD | Lemuriformes | RJWJ000000000.1 |
| *Cheirogaleus medius* | Lesser dwarf lemur | WVFYSCCPTTPYLD | Lemuriformes | GCA_004024725.1 |
| *Microcebus murinus* | Grey mouse lemur | WVFYSCCPTTPYLD | Lemuriformes | ENSMICT00000067396.1 |
| *Mirza zaza* | Northern giant mouse lemur | WVFYSCCPTTPYLD | Lemuriformes | VSMD00000000.1 |
| *Eulemur flavifrons* | Blue-eyed black lemur (Sclater's lemur) | WVFYSCCPNTPYLD | Lemuriformes | LGHW00000000.1 |
| *Saguinus imperator* | Emperor tamarin | WVFYSCCPDTPYLD | Platyrrhini | PVHO000000000.1 |
| *Callithrix jacchus* | White-tufted-ear marmoset | WVFYSCCPDTPYLD | Platyrrhini | U3E8V3 |
| *Aotus nancymaae* | Ma's night monkey | WVFYSCCPDTPYLD | Platyrrhini | A0A2K5CH45 |
| *Saimiri boliviensis* | Bolivian squirrel monkey | WVFYSCCPDTPYLD | Platyrrhini | A0A2K6V5P4 |
| *Cebus capucinus* | Colombian white-faced capuchin | WVFYSCCPDTPYLD | Platyrrhini | A0A2K5RK82 |
| *Sapajus apella* | Tufted capuchin | WVFYSCCPDTPYLD | Platyrrhini | WRPQ00000000.1 |
| *Alouatta palliata* | Mantled howler monkey | WVFYSCCPDTPYLD | Platyrrhini | PVKV000000000.1 |
| *Ateles geoffroyi* | Black-headed spider monkey | WVFYSCCPDTPYLD | Platyrrhini | PVHS00000000.1 |
| *Mandrillus leucophaeus* | Mandrill | WVFYSCCPNTPYLD | Cercopithecidae | A0A2K6AD85 |
| *Cercocebus atys* | Sooty mangabey | WVFYSCCPNTPYLD | Cercopithecidae | A0A2K5LJ67 |
| *Papio anubis* | Olive baboon | WVFYSCCPNTPYLD | Cercopithecidae | A0A096MY80 |
| *Theropithecus gelada* | Gelada | WVFYSCCPNTPYLD | Cercopithecidae | QGDE00000000.1 |
| *Macaca mulatta* | Rhesus monkey | WVFYSCCPNTPYLD | Cercopithecidae | F7ET74 |
| *Erythrocebus patas* | Patas monkey | WVFYSCCPNTPYLD | Cercopithecidae | PVJV000000000.1 |
| *Chlorocebus sabaeus* | Green monkey | WVFYSCCPNTPYLD | Cercopithecidae | AQIB00000000.1 |
| *Cercopithecus neglectus* | De Brazza's monkey | WVFYSCCPNTPYLD | Cercopithecidae | PVKI000000000.1 |
| *Rhinopithecus bieti* | Black snub-nosed monkey | WVFYSCCPNTPYLD | Cercopithecidae | A0A2K6K3B8 |
| *Pygathrix nemaeus* | Red-shanked douc | WVFYSCCPNTPYLD | Cercopithecidae | PVHW000000000.1 |
| *Semnopithecus entellus* | Northern plains grey langur | WVFYSCCPNTPYLD | Cercopithecidae | PVII000000000.1 |
| *Trachypithecus francoisi* | Francois' langur | WVFYSCCPNTPYLD | Cercopithecidae | GCA_009764315.1 & GCA_009764325.1 |
| *Piliocolobus tephrosceles* | Ugandan red colobus | WVFYSCCPNTPYLD | Cercopithecidae | PDMG00000000.3 |
| *Colobus angolensis* | Angolan colobus | WVFYSCCPNTPYLD | Cercopithecidae | A0A2K5HLD5 |
| *Hylobates moloch* | Silvery gibbon | WVFYSCCPNTPYLD | Cercopithecidae | WKKJ00000000.2 |
| *Nomascus leucogenys* | Northern white-cheeked gibbon | WVFYSCCPNTPYLD | Cercopithecidae | G1R1T5 |
| *Gorilla gorilla* | Western gorilla | SVTYSCCPDTPYLD | Homininae | GCA_008122165.1 |
| *Pan troglodytes* | Chimpanzee | SVTYSCCPDTPYLD | Homininae | H2QJ05 |
| *Homo sapiens* | Human | SVTYSCCPDTPYLD | Homininae | G5E9G9 |
| *Pongo abelii* | Sumatran orangutan | WVSYSCCPNTPYLD | Ponginae | H2P7W2 |
| *Carlito syrichta* | Philippine tarsier | WVFYSCCPNTPYLD | Tarsiiformes | A0A1U7TLB3 |
| *Nycticebus coucang* | Sunda slow loris | NVTYSCC-DTIYLD | Lorisiformes | PVIV010015948.1 |
